# Unlocking grass leaf development: foundations for tunable cereal design

**DOI:** 10.1111/nph.70477

**Published:** 2025-09-27

**Authors:** Trisha McAllister, Hilde Nelissen, Josh Strable, Annis E. Richardson

**Affiliations:** ^1^ Institute of Molecular Plant Science, School of Biological Sciences University of Edinburgh Kings Buildings Campus, Daniel Rutherford Building, Max Born Crescent Edinburgh EH9 3BF UK; ^2^ Department of Plant Biotechnology and Bioinformatics Ghent University 9052 Ghent Belgium; ^3^ VIB Center for Plant Systems Biology Technologiepark 71 9052 Ghent Belgium; ^4^ Department of Genetics, Development and Cell Biology Iowa State University Ames IA 50011 USA

**Keywords:** cereals, developmental biology, genetics, leaf development, plant engineering, plant science, programmable plants

## Abstract

The grass leaf plays a critical role in global food security, generating the carbon stores in cereal grains, which provide > 50% of global calories. As the global population grows, there is an urgent need to increase food production using fewer resources and to develop more resilient agricultural systems to withstand variable climate conditions and rising socio‐economic and environmental costs. Precision engineering of cereal crops, tailored to diverse environmental conditions and agronomic practices, is a vital strategy for achieving food security. Given the fundamental importance of the leaf in driving cereal productivity, it is an ideal engineering target. Leaf development occurs over large temporal and spatial scales and is environmentally regulated, posing significant challenges for predictive engineering approaches and limiting the feasibility of a one‐size‐fits‐all approach. In this review, we synthesise current understanding of cereal leaf development and identify critical developmental biology questions that must be resolved to facilitate the truly programmable plants of the future.

**Table:** 

	Contents	
	Summary	1655
I.	Introduction	1655
II.	Building a grass leaf	1656
III.	Formation of specialised structures and cell types	1665
IV.	Engineering a grass leaf	1669
	Acknowledgements	1670
	References	1670

## I. Introduction

Around 10 000 years ago, harvesting the seeds of a handful of grass species by humans changed the world. Modern descendants of these grass species, collectively called the cereals, provide > 50% of global calories. Our dominant cereal crops – wheat, rice, and maize – are members of diverse clades of the monophyletic grass family. Collectively, greater than 2500 million tons of cereal grain are harvested each year for both human and animal consumption (‘FAO Cereal Supply and Demand Brief’, 2024), and, increasingly, cereal biomass is being used as feedstock for renewable energy sources. Grass leaves are the indispensable powerhouse behind this incredible productivity.

**Fig. 1:**
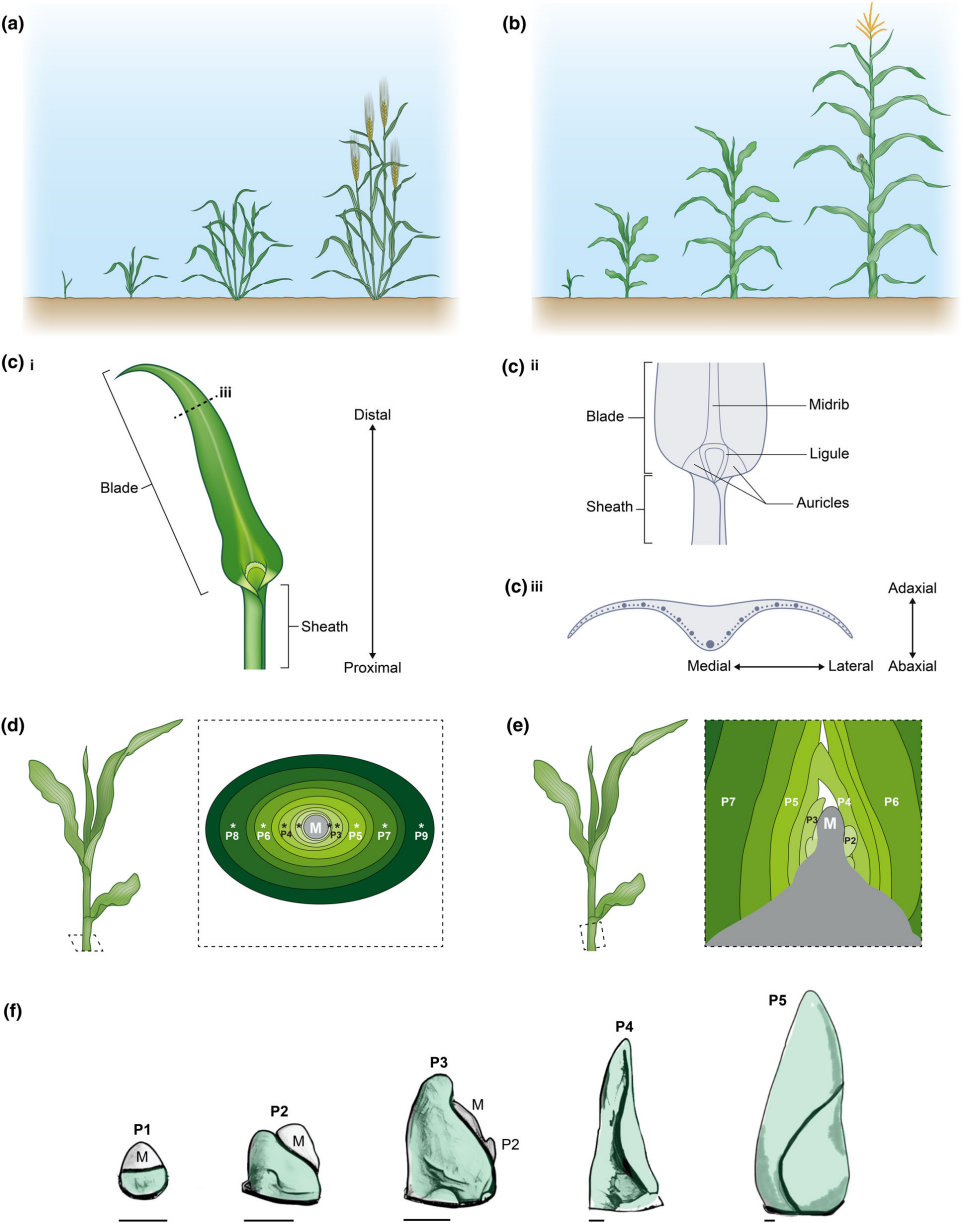
Cereal leaf development. Cartoon of cereal development in barley (*Hordeum vulgare*) (a) and maize (*Zea mays*) (b), illustrating the changes in vegetative architecture as the plant grows, and the differences in leaf size, shape, and angle across the lifespan of the plant. (c) The cereal leaf has distinct tissues along the proximal–distal axis (i & ii), and across the adaxial–abaxial and medial–lateral axes (iii). (d, e) Leaves encircle the meristem and all younger leaves; as such, single cross‐sections reveal multiple developmental stages of leaf development. (f) Leaves progress through a series of shape changes during early primordium development (green = primordium). Bars, 100 μm. P#, plastochron; M, shoot apical meristem. *, midvein.

The grass leaf has a distinctive, semi‐3D structure. The leaf base, called the sheath, wraps around younger leaves and stem, providing structural support and accounting for nearly all vertical height of the shoot during its vegetative phase. The upper part of the leaf, the blade, bends away from the main axis of the plant to intercept light. At the boundary between the sheath and blade, an adaxial fringe – called the ligule – forms to serve as a sliding gasket. At the leaf margins near the ligule, two triangular structures form – called auricles – and serve to regulate blade angle (Fig. 1a–c). Formation of the grass leaf requires precise coordination of genetic networks and growth across three geometric axes – adaxial–abaxial, medial–lateral, and proximal–distal – and developmental time (Fig. 1). Understanding how the grass leaf forms, the genetic networks that blueprint its development, and the environmental inputs that influence its growth to regulate its shape and size are of major agronomic interest. With recent rapid advances in genomics, imaging, bioengineering, and computational modelling, our understanding of cereal leaf growth and development has come forward in leaps and bounds, building on the solid foundation of decades of developmental genetic studies (Supporting Information Table S1), primarily in maize, rice, and barley (Kurata *et al*., 2005; Richardson & Hake, 2022; Hansson *et al*., 2024; Lorenzo *et al*., 2024).

Other reviews serve as excellent resources on additional aspects of leaf development (Conklin *et al*., 2019; Satterlee & Scanlon, 2019; Xu *et al*., 2021; Cao *et al*., 2022; Perico *et al*., 2022; Gong *et al*., 2024; Zhang *et al*., 2024). In this review, we synthesise our current understanding of cereal leaf development and highlight key outstanding questions, which may now be possible to address with recent technological advances. Ultimately, we ask an urgent overarching question: what do we need to understand to be able to precisely engineer grass leaf development for optimised productivity and quality in specific environments, agronomic practices, and end uses?

## II. Building a grass leaf

The grass leaf begins as a ring‐shaped collection of cells encircling the meristem (Sharman, 1942). Through the combined activity of patterning (e.g. gene expression and hormone patterns), cell dynamics (e.g. cell expansion and division), and tissue mechanics (e.g. tissue deformation and buckling), the shape of the cereal leaf arises over developmental time, progressing through a series of defined shape transitions (Fig. 1d–f). Each component – patterning, cell dynamics, and tissue mechanics – integrates both developmental and environmental signals, feeding back on each other to generate a robust leaf shape. Optimising each component over developmental time and space is crucial to determining final output. As such, to attain the goal of predictable and precise engineering of grass leaves, a comprehensive understanding of their development over a plant's lifespan, from initiation to final shape and structure, is urgent.

### 1. Grass leaf initiation

In flowering plants, organs like leaves are iteratively initiated on the flanks of the shoot apical meristem (SAM). Control of the number, timing, and spatial distribution of initiating organs (each referred to as a primordium) defines their spatial distribution around the stem and, therefore, contributes to mature plant architecture. The shape of the initiating primordium also has a significant impact on final organ shape (Scanlon, 2003; Nardmann *et al*., 2004; Richardson *et al*., 2020). In the case of leaves, these traits impact light interception and photosynthetic efficiency of the plant. The process of organ initiation is best understood in the eudicot model plant *Arabidopsis thaliana* (At), in which auxin accumulation, controlled by the activity of the auxin transporter AtPINFORMED1 (PIN1), is the first marker of incipient primordium formation. This is rapidly followed by downregulation of Class‐I *KNOTTED‐LIKE HOMEOBOX* (*KNOX*) genes and subsequent upregulation of leaf identity genes. Ultimately, this leads to the outgrowth of small crescent‐shaped primordia on the flanks of the SAM in a spiral phyllotactic pattern (reviewed in detail by Ali *et al*., 2020; Lv *et al*., 2023). All flowering plants share a common evolutionary origin of leaves (Boyce, 2010); therefore, it is likely several core mechanisms are shared between Arabidopsis and grasses, despite diverging *c*. 150 million years ago (Chaw *et al*., 2004). However, a key difference is the shape of the incipient primordium: all grasses have a ring‐shaped primordium that encircles the SAM (Richardson *et al*., 2021) (Fig. 1d–f). If we aim to optimise the timing and position of leaf formation in cereal crops, the differences unique to the grasses, and indeed, different species of grass, are crucial to understand through comparative analyses.

#### Recruitment of incipient leaf cells in grasses

Like in eudicot species, auxin signalling plays a central role during grass leaf initiation. Treatment of maize meristems with the auxin transport inhibitor N‐1‐naphthylphthalamic acid (NPA), for example, inhibits leaf initiation (Scanlon, 2003). However, there are key differences in this process between grass and eudicot plants. In grasses, there are three proteins closely related to AtPIN1 (O'Connor *et al*., 2014): PIN1a, PIN1b, and SISTER OF PINFORMED1 (SoPIN1). Each PIN1 protein has a clear sub‐function in the grass SAM (O'Connor *et al*., 2014, 2017). Crucially, formation of the epidermal auxin maxima required for leaf initiation is regulated by polarly localised SoPIN1 (O'Connor *et al*., 2017). An internal, incipient vascular strand forms just below this maxima through the activity of PIN1a and PIN1b, directing and refining regions of auxin flux to connect the epidermal maxima to the existing vasculature of the stem below (O'Connor *et al*., 2014). Ultimately, this incipient vascular strand will form the midvein of the leaf. Auxin transcriptional response then likely leads to reinforcement of this initial patterning, evidenced by mutants, such as the maize dominant auxin/indole‐3‐acetic acid (AUX/IAA) repressor mutant *Hoja loca1* (*Oja1*), which fails to initiate leaves and, when leaves are initiated, they often lack midribs (Richardson *et al*., 2020).

*KNOX* downregulation is concomitant with auxin maxima formation and, unlike in eudicot plants, occurs in a domain that completely encircles the meristem to form a ring‐shaped incipient primordium (P0) that includes both surface (L1) and internal (L2) meristem cells (Jackson *et al*., 1994). Clonal sector analyses in maize reveal that this P0 gives rise to all tissues in the leaf (Poethig, 1984). *KNOX* downregulation may involve both transcriptional and epigenetic silencing, as disruption of PHANTASTICA‐mediated epigenetic silencing in the maize *roughsheath2* (*rs2*) mutant results in leaf initiation defects (Schneeberger *et al*., 1998; Timmermans *et al*., 1999; Phelps‐Durr *et al*., 2005). Precise downregulation of *KNOX* is important for cell recruitment into the P0 and has key consequences for final leaf shape. WUSCHEL‐LIKE HOMEOBOX3 (WOX3) is an important player in this *KNOX* repression, promoting lateral expansion of the P0 around the SAM. In *wox3* mutants, such as maize *narrowsheath1*/*ns2* (*ns1*/*ns2*) and *ns1*/*ns2*/*wox3a*, barley *narrow leafed dwarf1* (*nld1*) and rice *narrow leaf2*/*nal3* (*nal2*/*3*), a failure to downregulate *KNOX* genes in the lateral bounds of the founder cells results in a deletion of the leaf margins and subsequent narrow leaf phenotypes (Scanlon *et al*., 1996; Nardmann *et al*., 2004; Cho *et al*., 2013; Yoshikawa *et al*., 2016; Satterlee *et al*., 2023).

*KNOX* downregulation is restricted to a specific region that limits cell differentiation to the P0 and maintains the stem cell population, delimiting a boundary that is also defined by the activation of *CUP‐SHAPED‐COTYLEDON* (*CUC*) genes. *CUC* expression bounds the P0 and defects in boundary formation result in fused leaves and meristem failure (Chang *et al*., 2021; Richardson *et al*., 2021; J. Wang *et al*., 2021; Han *et al*., 2023). In eudicot plants, the meristem‐organ boundary has distinctive patterns of hormone accumulation and/or response, gene expression, cell behaviour, and mechanics (Conklin *et al*., 2019; Nakayama *et al*., 2022). This is yet to be fully explored in grasses given the inaccessibility of the meristem (Fig. 1d,e); however, computational models suggest that differences in growth rates between the P0 and boundary region are also important for enabling the emergence of the leaf primordium (Chang *et al*., 2021; Richardson *et al*., 2021).

#### Emergence of the grass leaf primordium

**Fig. 2:**
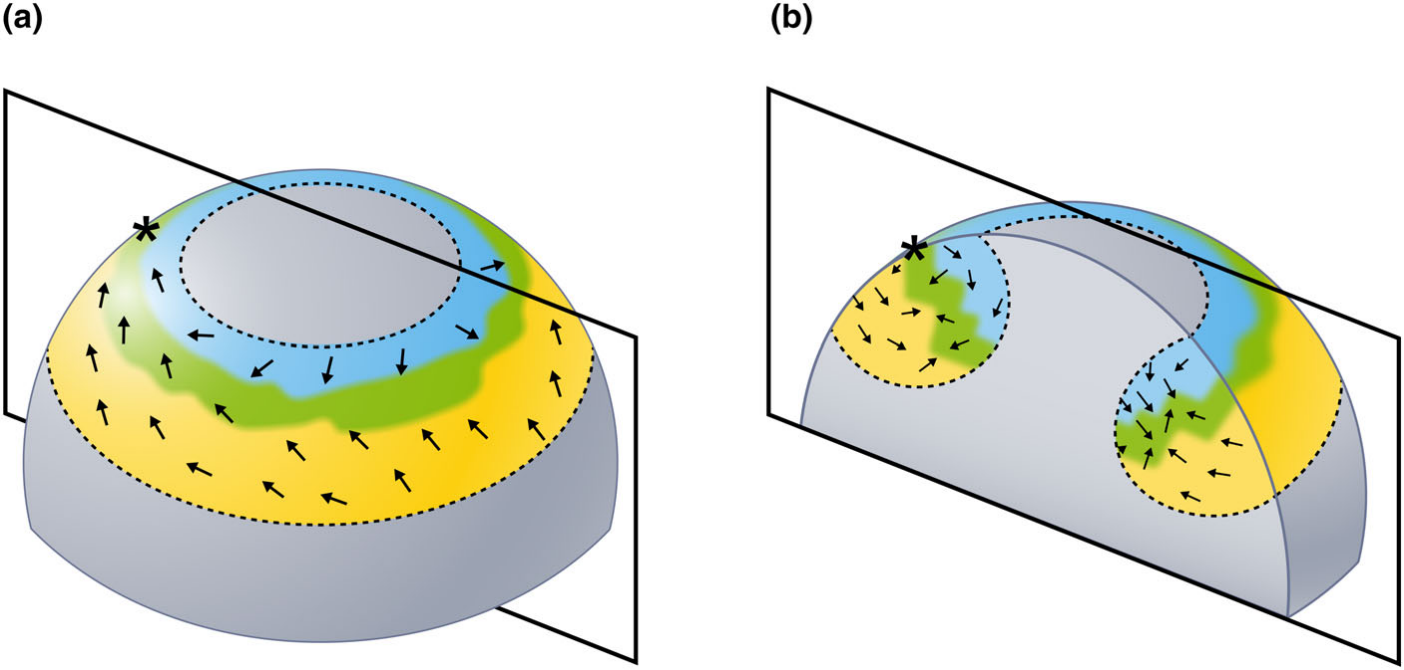
Illustration of processes involved in leaf primordium emergence. (a, b) Cartoons of a cereal vegetative meristem before the leaf primordium has emerged from the surface. The region of *KNOTTED‐LIKE HOMEOBOX* (*KNOX*) gene downregulation (Plastochron 0, P0) is outlined by a dotted line. Abaxial (yellow) and adaxial (blue) identity, and the boundary between the two (green), and the SoPIN1 convergence point (*) within the P0 are indicated. (a) A simplified illustration of the orientation of planar polarity (arrows) within the P0. (b) A cross‐section through (a) (boxed region) and a simplified illustration of *KNOX* expression in the meristem (grey shading), and orthoplanar polarity (arrows) in the P0 .

During emergence, or outgrowth, of the primordium, a wave of cell division and expansion spreads around the meristem circumference, resulting in the base of the leaf encircling the meristem and the formation of a ring‐shaped leaf primordium (Plastochron 1, P1) (Sharman, 1942) (Figs 1f, 2a). Computational modelling of gene expression and growth rate patterns required for grass leaf emergence (Richardson *et al*., 2021) predicts that two polarity axes are required: an orthoplanar axis that is orthogonal to the surface of the meristem (Fig. 2b), and a planar axis that is parallel with the surface (Fig. 2a). In the model, the orthoplanar polarity re‐orients in the P0 towards the boundary between the adaxial (Ad) and abaxial (Ab) identities, most likely ‘inherited’ from a prepattern laid down in the SAM (Juarez *et al*., 2004; Burian *et al*., 2022). Planar polarity orients away from the boundary of the P0 towards the auxin maxima and is based upon observed SoPIN1 localisation patterns. Combined, these two axes enable cells to specify growth rates in three orientations, facilitating emergence from the surface of the meristem (Richardson *et al*., 2021). Underlying these crucial growth rate and orientation differences may be *WOX* activity. At the Ad‐Ab boundary, *WOX3* activity is localised to a region congruous with the modelled ‘rim domain’ that will ultimately form the outer edges of the leaf (Richardson *et al*., 2021; Satterlee *et al*., 2023). These observations are consistent with the hypothesis that *WOX* genes promote laminar outgrowth and leaf primordium emergence across flowering plants, although the precise patterns of expression differ, corresponding to different primordium shapes (Lin *et al*., 2013; Andrejek *et al*., 2024).

Upon emergence from the meristem surface, grass leaf primordia progress through a series of distinct shape transitions (Fig. 1f), likely underpinned by asymmetrically distributed growth rates, varying from the midvein to the margin of the primordium (Richardson *et al*., 2021). Computational models show that, without this initial asymmetry, a tube‐shaped leaf will be formed. This predicted phenotype is reminiscent of disrupted auxin transcriptional responses seen in maize SAMs treated with NPA and in the *Oja1* mutant (Scanlon, 2003; Richardson *et al*., 2020), suggesting an intricate association between auxin and growth asymmetry. Direct measurements of growth rates in these early primordia have yet to be carried out, but model predictions are consistent with both mutant phenotypes and clonal sectors observed in mature leaves (Richardson *et al*., 2021). These models predict that small changes in shape during leaf initiation can result in dramatic changes in mature leaf shape, highlighting the crucial role of understanding the fundamental early steps in leaf formation for engineering purposes.

#### Iteration and positioning of grass leaf primordia

Organ initiation does not occur in isolation, but instead in a dynamic environment with an incipient primordium forming simultaneously with the emergence of the most recently formed primordium from the surface of the meristem. Precise timing and spatial distribution of these iteratively formed primordia result in different patterns of leaf formation that ultimately shape vegetative plant architecture and influence light interception efficiency.

The time between successive leaf primordium initiation (or P0 formation) is defined as a plastochron. Relatively little is known about the molecular networks involved in the temporal regulation of grass leaf initiation. Mutant analyses in rice, barley, and maize have identified several genes involved in regulating the plastochron, although they often exhibit other pleiotropic phenotypes. The plastochron is shortened in mutants like rice *plastochron1* (*pla1*), *pla2*, *pla3*, and *aberrant panicle organisation1* (*apo1*) (Itoh *et al*., 1998; Miyoshi *et al*., 2004; Ikeda *et al*., 2005, 2007; Kawakatsu *et al*., 2006, 2009), barley *many noded dwarf1* (*mnd1*), *mnd4*, *mnd8* (Hibara *et al*., 2021; Guo *et al*., 2023), and maize *terminal ear1* (*te1*) (Veit *et al*., 1998), *Truffula* (*Trf*) (Prigge *et al*., 2025), *big embryo1* (*bige1*) (Suzuki *et al*., 2015), and *viviparous8* (*vp8*) (Evans & Poethig, 1997), resulting in dramatic increases in leaf number, smaller leaves, and aberrant plant architecture. Conversely, maize *phyB1;phyB2* double mutants have longer plastochrons, resulting in fewer leaves and longer internodes (Sheehan *et al*., 2007). These mutants represent defects in cytochrome P450 78As (*pla1*, *mnd4*), MEI2‐like RNA binding proteins (*pla2*, *te1*), glutamate carboxypeptidases (*pla3*, *vp8*), MATE transporters (*mnd8*, *bige1*), phytochrome receptors (*phyB1;phyB2*), N‐acetyltransferases (*mnd1*), F‐box proteins (*apo1*), and auxin response factors (*Trf*). Comparative and double mutant analyses so far suggest that many of the genes identified likely act in separate pathways (Kawakatsu *et al*., 2006; Hibara *et al*., 2021; Busche *et al*., 2023), indicating complex regulation of the plastochron.

Despite the observed complexity, multiple hypotheses have been posed to explain the temporal regulation of leaf initiation in grasses. These hypotheses are derived from the analysis of cell behaviours, gene expression patterns, and leaf morphology. One hypothesis proposes that cell division rates may determine the plastochron, due to its strong correlation with changes in the plastochron in rice *pla1* (Itoh *et al*., 1998). Alternatively, the plastochron may be defined by a (yet unknown) noncell autonomous signal produced by leaf primordia that inhibits P0 formation in the meristem and whose strength/efficacy declines as the leaf matures – a hypothesis based on the observation that some plastochron mutants have accelerated leaf maturation yet the expression of associated genes is outside the meristem (Kawakatsu *et al*., 2006). In rice, the link between gibberellic acid (GA) signalling and *PLA1* and *PLA2* expression is posited as support for such a noncell autonomous signal, as GA promotes leaf development, yet GA also promotes cell division and the expression of *PLA1* and *PLA2* (Mimura *et al*., 2012). More recently, high‐resolution spatial gene expression analyses using an *in situ* sequencing (ISS) technology ties these two hypotheses together. ISS in maize shoot apices revealed a precise expression pattern of *PLA1*, a direct target of KNOTTED1 (KN1; Bolduc *et al*., 2012), subtending the leaf primordium in a domain between undifferentiated and differentiated cell populations. As such, maize *PLA1* may be involved in regulating the balance between differentiated and undifferentiated cells in the meristem, potentially through interacting with auxin and cytokinin (CK) signalling (Laureyns *et al*., 2022). By inhibiting differentiation, PLA1 may act locally to limit the capacity for the initiation of new primordia. When *PLA1* is knocked out in maize, this inhibition is released, shortening the plastochron. A role for *PLA1* in impeding differentiation is further supported by the observation that enhanced expression of *PLA1* in the base of the maize leaf results in larger leaves by prolonging the duration of basal cell division (Sun *et al*., 2017). The enzymatic product of *PLA1* and the mechanism for its action remain unknown. Perhaps the crucial role of the ratio of undifferentiated to differentiated cell populations may explain why so many independent regulators of the plastochron have been identified and why these genes have pleiotropic effects.

While the temporal regulation of leaf initiation determines the rate of leaf formation, the spatial regulation of where the next leaf primordium (P0) is initiated defines the arrangement of leaves, or phyllotaxis, around the stem of the mature plant. Phyllotactic patterns are varied across land plants, with spiral phyllotaxy dominating in flowering plants. However, grasses differ from this ‘norm’ as all grasses (and many other monocots) share a distichous pattern of leaf arrangement (Barnard, 1964). Each grass P0 forms opposite the previous auxin maxima position by 180 degrees (Fig. 1d). Current models of phyllotactic patterns are eudicot‐centric and hinge on essential roles of auxin dynamics and/or tissue mechanics in establishing the position of the P0 (e.g. reviewed by Reinhardt & Gola, 2022). Given the single origin of leaves in flowering plants, it is likely that grass phyllotaxy involves a similar process described by eudicot models; nonetheless, further research focusing on grass phyllotaxy remains important.

In grasses, remarkably few mutants have defects in leaf phyllotaxy. Maize mutants *te1* (Veit *et al*., 1998), *Trf* (Prigge *et al*., 2025), and *aberrant phyllotaxy1* (*abph1*) (Jackson & Hake, 1999), and rice mutants *decussate* (Itoh *et al*., 2012) and *shoot organisation1* (*sho1*) (Itoh *et al*., 2000) deviate from the normal distichous phyllotactic pattern. These mutants implicate CK and auxin signalling, as well as stem cell regulation in directing spatial patterns of leaf emergence. Interestingly, analysis of meristem size and shape in *abph1*, which can revert spontaneously to distichous phyllotaxy, and in *sho1* suggests that grass phyllotaxy changes are a consequence of both meristem size and shape. This is further supported by the observation that meristem size varies greatly across maize lines (Leiboff *et al*., 2015), whereas phyllotaxy appears rather stable. The paucity of phyllotaxy mutants in grasses suggests that the spatial regulation of leaf initiation may be tightly regulated and/or developmentally constrained. Interestingly, grass inflorescence architecture, a related character, is more varied (Bommert & Whipple, 2018), raising the possibility that leaf arrangement around the stem could be altered by engineering new genetic modules or tweaking endogenous pathways.

A major challenge is to modulate leaf initiation, either spatially or temporally, to optimise plant architecture for different environmental and agronomic conditions. The highly pleiotropic role of many of the genes identified to date, combined with an overall poor understanding of the spatial and temporal regulation of grass leaf initiation, limits our ability to precisely modulate genetic networks controlling plant architecture. For example, many of the plastochron mutants identified to date have altered flowering times. In some cases, such traits can be genetically separated, as with mutations in *TRF* and *VP8* that do not alter flowering time compared with their normal siblings (Evans & Poethig, 1997). Interestingly, leaf azimuthal angle shows strong gene‐by‐environment effects in the field and modifies performance in dense planting environments (Zhou *et al*., 2024). Whether leaf azimuthal angle is due to subtle differences in meristem size and shape, phyllotaxy deviations, or compensatory growth in the leaves remains to be reported. An increased understanding of the networks underpinning leaf initiation and positioning carries significant potential for unlocking our ability to fine‐tune vegetative development in cereal crops. Knowledge around this topic will benefit particularly from recent advances in live imaging, transformation and regeneration (Chen *et al*., 2022), single‐cell/nuclei transcriptomics and genomics (Seyfferth *et al*., 2021; Minow *et al*., 2023) and spatial transcriptomic approaches (Yu *et al*., 2023; Bawa *et al*., 2024).

### 2. Grass leaf patterning

Analysis of mutants with altered mature leaf shapes has revealed that the patterns established across the three axes – abaxial–adaxial, medial–lateral, and proximal–distal–during the earliest phases of leaf development (Fig. 1c) are crucial for producing the final leaf shape and the structures important for function. Recent studies using comparative genetics, computational modelling, single‐cell transcriptomics, and spatial expression techniques have generated key insights into the underlying genetic patterns critical to early stages of leaf development.

#### Abaxial–adaxial patterning

**Fig. 3:**
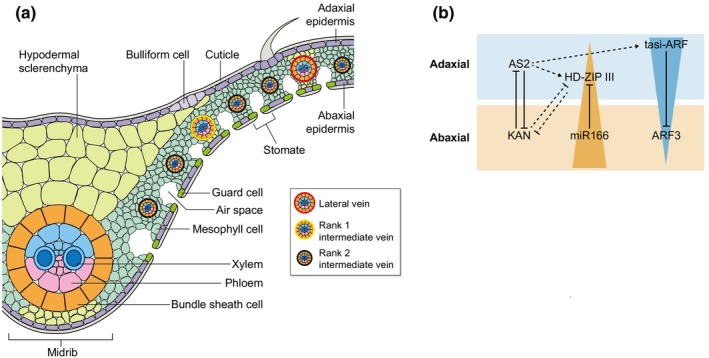
Adaxial–abaxial patterns in the grass leaf. (a) A cartoon depicting the distribution of different cell types across the adaxial–abaxial (Ad‐Ab) axis in a maize (*Zea mays*) leaf. (b) A summary of the key components of Ad‐Ab patterning conserved in grasses. AS2: ASYMMETRIC LEAVES2; KAN: KANADI; miR: microRNA; ARF: AUXIN RESPONSE FACTOR; HD‐ZIP III: class III homeodomain‐leucine zipper; tasiR: trans acting small interfering RNA.

Differentiation of the upper (adaxial, ‘Ad’) and lower (abaxial, ‘Ab’) surfaces of the leaf is crucial for leaf formation and function. Juxtaposition of Ad‐Ab identities in the developing leaf facilitates flat, laminar outgrowth (Waites & Hudson, 1995; Juarez *et al*., 2004). Conversely, ectopic expression of genes involved in Ad‐Ab patterning results in novel outgrowths in both eudicots and monocots (e.g. Waites & Hudson, 1995; Timmermans *et al*., 1998; Eshed *et al*., 2001, 2004; Emery *et al*., 2003; Candela *et al*., 2008; Dotto *et al*., 2014). Even though a typical grass leaf is far less differentiated along the Ad‐Ab axis compared with leaves of eudicot plants, asymmetric distribution of tissues and cell types across that axis in the grass leaf has key functional roles and influences response to environmental stimuli (Fig. 3a). For example, specialised structures, such as the ligule (discussed in detail below), are found on the adaxial surface, as well as adaxial vacuolated achlorophyllous parenchyma cells over the midrib in maize leaves that support the blade and influence leaf angle (Strable *et al*., 2017). Asymmetric patterning of mesophyll and bundle sheath cells along the Ad‐Ab axis is also linked with the development and function of specialised vascular structures important for C_4_ photosynthesis (Bezrutczyk *et al*., 2021) – a major target in C_3_ cereal engineering projects (Sedelnikova *et al*., 2018). Leaf rolling, driven largely by the deformation or shrinkage of adaxial bulliform cells (Kadioglu & Terzi, 2007), is a key response to heat and water stress. Natural diversity in bulliform cell morphology and distribution characterised by high‐throughput machine learning models (Qiao *et al*., 2019) correlates with differences in leaf rolling rates and therefore potential environmental resilience (Matschi *et al*., 2020). Such diverse Ad‐Ab patterning within and across grass leaves highlights the importance of understanding the genetics underpinning these complex leaf surfaces.

In eudicots, a molecular prepattern of concentric rings of adaxial and abaxial progenitor cells in the SAM that express *ASYMMETRIC LEAVES2* (*AS2*) and *KANADI* (*KAN*), respectively, predictively patterns the Ad‐Ab axis in the leaf (Burian *et al*., 2022). This prepattern resolves into a polarised transcriptional response to auxin that is otherwise uniformly distributed in the P0, leading to distinctive Ad‐Ab delineation of transcription factors that programme specific cell fates, such as the Class III *HOMEODOMAIN‐LEUCINE ZIPPER* (*HD‐ZIP III*) and *KAN* genes (Burian *et al*., 2022). Consistent with a meristem prepattern, *HD‐ZIP III* orthologue *ROLLED LEAF1* (*RLD1*) transcripts accumulate adaxially in the grass meristem/P0 (Juarez *et al*., 2004; Nogueira *et al*., 2007, 2009). While maize *MILKWEED POD1* (*MWP1*) and rice *SHALLOT‐LIKE1* (*SLL1*) genes, both encoding KAN transcription factors, are expressed in the abaxial epidermis in older leaf primordia and throughout early‐stage primordia and vasculature (Candela *et al*., 2008; Zhang *et al*., 2009). Loss‐of‐function mutations in these genes result in the development of adaxial features in abaxial positions, suggesting a conservation of the Ad‐Ab specification mechanism, as well as a prolonged period of regulation required to ensure that the entire leaf exhibits normal Ad‐Ab axiality.

A core regulatory network in leaf Ad‐Ab patterning is the formation of two opposing gradients of small RNAs that negatively regulate the transcription factors that define adaxial (HD‐ZIP III) and abaxial (auxin response factors (ARFs)) identities (Andrejek *et al*., 2020). An abaxial gradient of miR166 inhibits HD‐ZIP III accumulation, and an adaxial gradient of *trans*‐activating short‐interfering RNAs (ta‐siRNA) inhibits ARF accumulation (Fig. 3b). Gain‐of‐function mutations in maize, wheat, and rice with altered Ad‐Ab patterning indicate a conserved role for these small RNA‐transcription factor interactions in grasses. For example, dominant HD‐ZIP III mutants with lesions in the miR166 target site, like maize *Rld1*, wheat *paired spikelet1* (*ps1*), and rice *lateral floret 1* (*lf1*), have leaves that roll inward due to ectopic abaxial patterning (Nelson *et al*., 2002; Juarez *et al*., 2004; Dixon *et al*., 2022); (Itoh *et al*., 2008; Zhang *et al*., 2017, 2021; N. Li *et al*., 2020). Overexpression of a short tandem target mimic inactivates miR166 in maize, or miR166‐resistant *OSHB* transgenes in rice both phenocopy the abaxialised mutants (Itoh *et al*., 2008; Zhang *et al*., 2017, 2021; N. Li *et al*., 2020). Mutations in maize and rice genes required for ta‐siRNA pathways also have varying effects on Ad‐Ab patterning. Maize *LEAFBLADELESS1* (*LBL1*) and rice *SHOOTLESS2* (*SHL2*), *SHL4*, and *SHO1* genes encode key components of the ta‐siRNA biogenesis pathway, and mutants of any of these genes have leaves that are abaxialised with radial symmetry. Mutants with strong alleles can be shootless, while weak alleles confer different degrees of leaf curling. Transgenic maize plants overexpressing a tasiR‐ARF‐insensitive *ARF3* transgene, also phenocopy aspects of *lbl1* mutant leaves (Timmermans *et al*., 1998; Nagasaki *et al*., 2007; Nogueira *et al*., 2007; Itoh *et al*., 2008; Dotto *et al*., 2014; Thompson *et al*., 2014; Petsch *et al*., 2015), demonstrating how transgenic manipulation of these conserved gradients can alter leaf morphology.

Beyond core Ad‐Ab patterning modules, regulation of some grass leaf features suggests divergent functions in key genes. Genetic interaction analyses in the leaf indicate that *mwp1* (KAN) and *Rld1* (HD‐ZIP III) interact synergistically, not antagonistically, with *RLD1* transcripts accumulating ectopically on the abaxial side of *mwp1* mutant primordia (Candela *et al*., 2008). Mutant analyses in rice also show a role for the CK receptor ADAXIAL‐ABAXIAL BIPOLAR LEAF1 in regulating leaf polarity (Tezuka *et al*., 2024). In addition, YABBY transcription factors in grasses appear to have diversified in function, and to date not all members of the clade have been functionally characterised. In particular, *CRABS CLAW* (*CRC*) members of the *YABBY* family do not appear to have a role in fundamentally patterning the Ad‐Ab axis, but instead underlie the differentiation of asymmetrically distributed cell types, such as hypodermal sclerenchyma cells in the midrib, whereas FILAMENTOUS clade members have roles in inflorescence development (Strable *et al*., 2017; Tanaka *et al*., 2017). Understanding these divergences in gene functions through comparative genetic analyses will be important to predictably engineer Ad‐Ab patterning in cereals.

#### Medial–lateral patterning

**Fig. 4:**
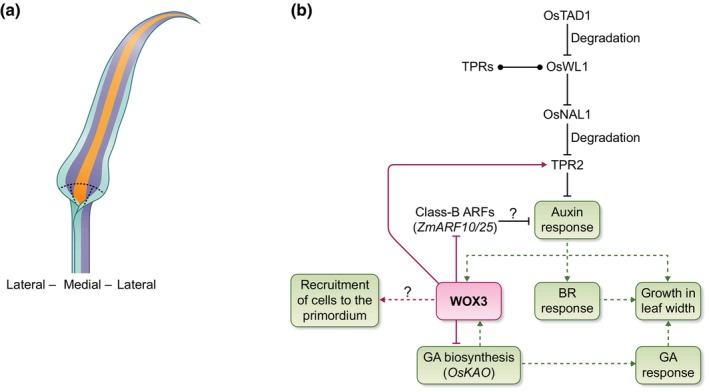
Medial–lateral patterning in the cereal leaf. (a) A cartoon illustrating potential different medial–lateral domains in the cereal leaf based on mutant phenotypes. (b) A summary overview of the regulation of medial–lateral patterning and growth in the cereal leaf. Dotted lines indicate indirect relationships. Solid lines indicate direct interactions or regulation. ‘?’ indicates relationships yet to be tested or understood. Arrows: positive regulatory interaction. Blunt ends: inhibitory regulatory interaction. Dotted ends: physical interaction between proteins. Os: *Oryza sativa*; Zm: *Zea mays*; BR: brassinosteroids; GA: gibberellic acid; WL1: WIDE LEAF1; TAD1: TILLERING AND DWARF1; TPR: TOPLESS‐RELATED PROTEIN; NAL1: NARROW LEAF1; ARF: AUXIN RESPONSE FACTOR; KAO: ENT‐KAURENOIC ACID OXIDASE; WOX3: WUSCHEL‐RELATED HOMEOBOX3.

Specification of the medial–lateral (M‐L) axis is responsible for recruiting cells into the ring primordium (discussed above), delimiting specialised structures in the leaf and contributing to leaf width. The maize leaf provides a particularly clear example of M‐L patterning with the formation of the prominent midrib in the central domain, and specialised saw‐tooth hairs on the leaf margin (Fig. 1). *WOX* genes have a prominent role in establishing domains across the M‐L axis (Fig. 4a), with mutants in multiple grass species showing defects in distinct M‐L domains (Scanlon & Freeling, 1997; Hay & Hake, 2004; Nardmann *et al*., 2004; Cho *et al*., 2013; Yoshikawa *et al*., 2016; Satterlee *et al*., 2023). RNA *in situ* hybridisation patterns and single‐cell RNA‐sequencing analysis revealed specific populations of cells with distinct *WOX* transcripts, which correspond to domains predicted by computational models and mutant analyses (Scanlon & Freeling, 1997; Hay & Hake, 2004; Nardmann *et al*., 2004; Cho *et al*., 2013; Yoshikawa *et al*., 2016; Conklin *et al*., 2020; Richardson *et al*., 2021; Satterlee *et al*., 2023). It is unknown how these distinct overlapping *WOX* expression domains are specified. However, unlike in Arabidopsis, current evidence suggests that highly specific spatial regulation in the most marginal cells of the developing leaf primordium is essential for correct leaf formation, as has been shown for maize *NS1* and *NS2* (Conklin *et al*., 2020). An additional layer of WOX activity regulation is revealed by the rice *narrowleaf21* (*nrl21*) mutant (Uzair *et al*., 2021). *nrl21* mutants are defective in the small ribosomal subunit RPS3A. RPS3A translationally regulates the *NS* homologue *WOX3a*, suggesting that modulation of both transcript levels and translation efficiency of *WOX* gene and gene products is important in regulating M‐L patterning and, ultimately, leaf width in cereals.

In addition to pre*cis*e *WOX* domain patterning, formation of the medial midrib requires activity of the YABBY transcription factor DROOPING LEAF (rice, DL, and maize, DRL, henceforth DL; Yamaguchi *et al*., 2004; Strable *et al*., 2017; Patterson *et al*., 2025). *DL* expression is one of the earliest leaf identity markers (Yamaguchi *et al*., 2004; Brooks *et al*., 2009) and is required for midrib formation in the blade (Yamaguchi *et al*., 2004; Strable *et al*., 2017; Patterson *et al*., 2025). This role of *DL* genes in promoting the proliferation of adaxial achlorophyllous parenchyma cells in the central domain of the leaf primordium appears to be unique to monocots (Strable *et al*., 2017). How DL acts, and whether other YABBY transcription factors, including those outside the *CRC* clade, function in leaf patterning in grasses is yet to be fully understood.

Leaf width is a complex quantitative trait and is impacted by both WOX‐mediated domain recruitment and growth (Scanlon & Freeling, 1997; Hay & Hake, 2004; Nardmann *et al*., 2004; Cho *et al*., 2013; Yoshikawa *et al*., 2016; Conklin *et al*., 2020; Richardson *et al*., 2021; Satterlee *et al*., 2023). Genes putatively downstream of NS1/NS2 reveal that *WOX3a* orthologues determine leaf width through regulating growth rates in the primordial leaf margin (Fig. 4b). Functioning downstream of the hormones CK and auxin, NS1/NS2 inhibit the expression of genes involved in cell division regulation, including the class‐B auxin response factors *ZmARF10* and *ZmARF25*, co‐orthologues of *AtARF2* (Conklin *et al*., 2020). AtARF2 has been shown to control organ size in Arabidopsis (Okushima *et al*., 2005; Schruff *et al*., 2006), and genetic interaction analyses revealed that AtARF2 activity is required for the *pressed flower*/*WOX3a* narrow leaf phenotype (Conklin *et al*., 2020). There are four AtARF2 orthologues in maize (*ZmARF10*, *ZmARF13*, *ZmARF25*, and *ZmARF28*) for which transcripts can be detected in the SAM and leaf primordia (Knauer *et al*., 2019; Conklin *et al*., 2020). Redundant functions of ZmARF10/13/25/28 are likely during leaf development; however, mutational analyses of these genes have yet to be reported. Interestingly, characterisation of the dominant *Trf* maize mutation in *ZmARF28* has uncovered diverse leaf size and shape defects due to ZmARF28 protein stabilisation (Prigge *et al*., 2025), although the relationship with NS1/NS2 is yet to be determined. Collectively, these observations indicate that regulation of transcript and protein levels should be considered carefully when outlining strategies to modulate leaf width in cereals.

Consistent with a close interaction between hormone signalling and *WOX*‐mediated laminar growth, mutant analysis of rice *WOX3a* highlights an important negative feedback loop with GA (Cho *et al*., 2016). The exact role GA plays in regulating leaf width is yet to be fully evaluated in maize; however, extensive analysis of GA's role in controlling the extent of the division zone in the leaf suggests that it may act as a key integrator of environmental signals, such as drought or cold, while also modulating leaf length (Nelissen *et al*., 2012; De Vos *et al*., 2020; Band *et al*., 2022). This opens a possibility for GA's involvement in environmental regulation of cereal leaf width via impinging on *WOX3a* activity.

Extensive analysis of leaf width mutants in rice and other cereals has revealed additional regulatory components, including key roles of hormone pathways. Unsurprisingly, auxin signalling plays a prominent role in controlling the degree of lateral growth. Rice ARF11 directly regulates brassinosteroid (BR) sensitivity through positive regulation of the BR receptor *BRASSINOSTEROID INSENSITIVE1* (Sakamoto *et al*., 2013), connecting two major hormone signalling pathways involved in growth and development. The rice *narrow leaf21* (*nal21*) mutant is less responsive to auxin through the translational regulation of *OsARF11 and OsARF16* (Uzair *et al*., 2021). Rice *nal7* has a mutation in a *YUCCA* auxin biosynthesis gene (Fujino *et al*., 2008; Qi *et al*., 2008), while *nal1* has a mutation in a putative trypsin‐like serine/cysteine protease (Fujino *et al*., 2008; Qi *et al*., 2008); each of these examples highlights key roles for auxin production and its transport in regulating leaf size. Rice *NAL1* is negatively regulated by rice WIDE LEAF1 (WL1), a zinc finger transcription factor that recruits TOPLESS‐RELATED (TPR) corepressors to the *NAL1* promoter (You *et al*., 2022). In turn, *WL1* is negatively regulated by rice TILLERING AND DWARF1, which promotes ubiquitination and degradation of WL1 via the 26S proteasome (You *et al*., 2022). Interestingly, recent work indicates that NAL1 is able to physically interact with TPR2, causing its degradation, thereby driving the upregulation of auxin‐ and strigolactone‐related genes (Li *et al*., 2023). Mutations in the barley *HIGH NUMBER OF TILLERS1* gene, an ortholog of rice *NAL1*, indicate that changes to protein–protein interactions may be crucial in determining overall leaf size (Jöst *et al*., 2024). The *SPIKE* allele of rice *NAL1* results in large panicles, broad flag leaves, and increased yields in rice (Fujita *et al*., 2013), demonstrating the key potential of tuning leaf width by modulating levels and activities of these proteins in a tissue‐ and/or time‐specific manner.

#### Proximo‐distal patterning

Upon emergence from the meristem, the leaf primordium has an inherent proximal–distal (P‐D) axis. As development progresses, cells differentiate along this axis across the sheath, auricle, ligule, and blade tissues. Patterning that delineates these distinctive leaf domains occurs during the earliest developmental stages of the primordium: by plastochron 6 (P6) the preligule band that marks the incipient ligule has emerged, and the cells along the P‐D axis have distinct morphological and mechanical properties (Sylvester *et al*., 1990; Neher *et al*., 2023). Computational models of leaf development suggest that the timing of sheath and blade specification is important for final leaf shape due to differential effects on growth rates (Richardson *et al*., 2021), suggesting that P‐D axis patterning is tightly regulated spatially and temporally.

**Fig. 5:**
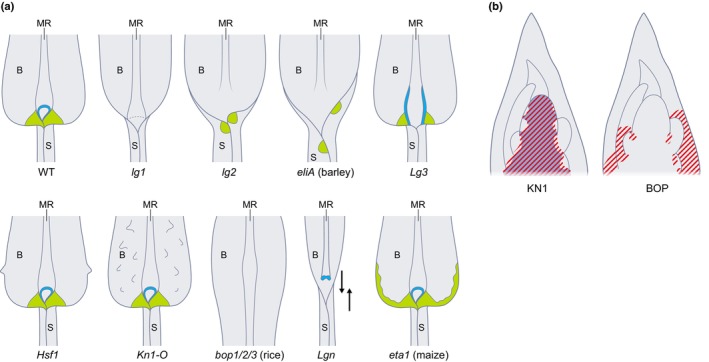
Proximal‐distal (P‐D) patterning mutants and the spatial patterns of known genetic regulators of P‐D patterning. (a) Cartoons of the leaf phenotype for mutants that have defects along the P‐D axis in maize (*Zea mays*), rice (*Oryza sativa*) and barley (*Hordeum vulgare*). WT: wild‐type, *liguleless1* (*lg1*), *liguleless2* (*lg2*), *eligulum A* (*eliA*), *Liguleless3* (*Lg3*), *Hairy sheath frayed* (*Hsf1*), *Knotted 1 ‐O* (*Kn1‐O*), *blade on petiole 1/2/3 triple mutant* (*bop1/2/3*), *Liguleless narrow* (*Lgn*), *extended auricle in blade* (*eta1*). S: sheath. B: blade. MR: midrib. Green: auricles. Blue: ligule. (b) mRNA (solid blue) and protein (red stripes) patterns for KNOTTED1 (KN1) and BLADE ON PETIOLE (BOP) based on RNA *in situ* hybridisation and immunolocalisation in maize. KN1 protein moves very locally distally into the base of the developing leaf primordium. BOP protein is in the proximal base of the developing leaf primordium.

Development of the P‐D axis requires regulation across four phases: (i) initiation of a new P‐D axis; (ii) specification of domain identities; (iii) maintenance of boundary domains during tissue growth and deformation; and (iv) regulation of morphological changes specific to each domain. Precise spatiotemporal regulation across all four stages is required to form a normal leaf. Patterning of the P‐D axis occurs through phases (ii) and (iii). Misregulation of genes involved in P‐D axis development can result in dramatic leaf morphology changes, such as ectopic leaf outgrowths (Smith *et al*., 1992; Lewis *et al*., 2014; Tavakol *et al*., 2015; Muszynski *et al*., 2020), altered leaf angle (Harper & Freeling, 1996; Walsh *et al*., 1998; Makarevitch *et al*., 2012; Wang *et al*., 2022), leaf shape (Moon *et al*., 2013; Satterlee *et al*., 2023), and perturbed sheath : blade ratio (Toriba *et al*., 2019) (Fig. 5a).

P‐D axis initiation (Phase i) is likely regulated by KNOX activity (Smith *et al*., 1992; Jackson *et al*., 1994; Ramirez *et al*., 2009). The KNOX stem cell regulator KN1 acts noncell autonomously and moves from meristem cells into basal leaf primordium cells, molecularly differentiating proximal leaf cells at initiation (Fig. 5b) (Hake & Freeling, 1986; Jackson, 2002). Given KN1's direct role in regulating the expression of key genes involved in the synthesis of, and the sensitivity of cells to, diverse hormones implicated in P‐D patterning, including GA, auxin, BR, and CK (Bolduc *et al*., 2012), it may be that KN1 triggers the formation of contrasting P‐D gradients of hormones in the initiating leaf primordium. Clear hormone gradients across the grass leaf P‐D axis are yet to be shown, but with the development of ratiometric reporters (Liao *et al*., 2015; Rizza *et al*., 2017; Dao *et al*., 2025) and spatial mass spectrometry (Petřík *et al*., 2024) this may soon be possible to test.

*KNOX* genes are also able to specify P‐D domains (Phase ii) in addition to, or as part of, their role in triggering P‐D axis formation. *KNOX* genes are sufficient to promote proximal identity (sheath) even when ectopically expressed in the blade (Smith *et al*., 1992; Foster *et al*., 1999; Ramirez *et al*., 2009; Satterlee *et al*., 2020; Leiboff *et al*., 2021). This observation suggests that proximal sheath identity is able to repress the potentially ‘default’ blade identity (Richardson *et al*., 2021). Consistent with this hypothesis, enhanced expression of *BLADE ON PETIOLE* (*BOP*) in rice increases the sheath:blade ratio (Dong *et al*., 2017; Toriba *et al*., 2019; Richardson *et al*., 2021; Wu *et al*., 2025). Indeed, developmentally regulated *BOP* transcript levels are proposed to be responsible for differences in sheath:blade ratio across leaves of different ages observed in rice (Dong *et al*., 2017; Toriba *et al*., 2019; Richardson *et al*., 2021; Wu *et al*., 2025). How BOP activity results in sheath identity is unclear. BOP proteins lack DNA binding domains (Hepworth *et al*., 2005), so it is conceivable they have a role as scaffolds that bring together diverse transcriptional regulators to control the expression of sheath identity genes. Transcriptional and chromatin immunoprecipitation (ChIP)‐seq analyses of maize reveal that *BOP* expression is directly downstream of KN1 (Bolduc *et al*., 2012; Leiboff *et al*., 2021), molecularly linking initiation of the P‐D axis (Phase i) and specification of P‐D domains (Phase ii). CK signalling mutants also form ectopic sheath‐like outgrowths in the blade (Muszynski *et al*., 2020), suggesting that, like *KNOX* genes, CK signalling also promotes proximal identity. Further investigation of KNOX and CK in P‐D patterning will likely reveal additional targets for dynamic modulation of sheath to blade ratio across grass species.

In contrast to the sheath, no genes that define distal (i.e. blade) identities have been found via mutant screens. Blade identity being the ‘default’ state could explain this, as knockouts of blade identity genes would be lethal. Alternatively, there may not be a specific blade fate, only ‘non‐sheath’. Accumulation of SoPIN1 in the distal tip of the incipient primordium (Richardson *et al*., 2021; Neher *et al*., 2023) suggests that auxin signalling may be involved in distal patterning, although how auxin signalling relates to distal identity is not known. Utiliising conditional mutants, or overexpression transgenic lines, may be essential for identification of blade specification factors. Alternatively, comparative analysis with homologous floral organs may enable identification of genes involved in blade identity regulation (Patterson *et al*., 2023; Richardson *et al*., 2024).

The boundary between the sheath and blade is demarcated, at least in part, by the noncell autonomous activity of the bZIP transcription factor LIGULELESS2 (LG2) (Harper & Freeling, 1996; Walsh *et al*., 1998). Maize and rice *lg2* mutants have a diffuse blade–sheath boundary and fail to form a complete ligule (Harper & Freeling, 1996; Walsh *et al*., 1998; R. Wang *et al*., 2021). Double mutant, gene expression, and DNA binding analyses show that maize LG2 is directly upstream of both BR signalling and *LIGULELESS1* (*LG1*), which drives ligule outgrowth (Phase iv) (Harper & Freeling, 1996; Walsh & Freeling, 1999; Hay & Hake, 2004; Moon *et al*., 2013; R. Wang *et al*., 2021). In Arabidopsis, BOP proteins have been shown to interact genetically and physically with PERIANTHIA, a bZIP protein in the same clade as LG2, suggesting a conserved interaction could exist in the grasses (Jakoby *et al*., 2002; Hepworth *et al*., 2005). If such interactions occur in the grasses, it may be that LG2 interacts physically with BOP, which would link proximal patterning and boundary specification (Richardson & Hake, 2018). Loss‐of‐function *bop* mutations in barley and brachypodium result in disrupted boundary specification and loss of ligules, supporting a potential role of BOPs in the blade–sheath boundary specification mechanism (Tavakol *et al*., 2015; S. Liu *et al*., 2022). Analysis of other mutants with diffuse or disrupted blade–sheath boundaries implicates roles for RNase‐H domain proteins (encoded by barley *ELIGULUM‐A*, *HvELI‐A* (Okagaki *et al*., 2018)) and serine–threonine kinases (maize *LIGULELESS NARROW*, *LGN* (Moon *et al*., 2013)) in boundary specification or maintenance, perhaps through regulation of transcription and protein phosphorylation, respectively. However, the precise roles of *HvELI‐A* and *LGN*, and how they may interact with *LG2*, are unknown. The maize mutant *extended auricle in blade1* (*eta1*) displaces auricles into the blade at the leaf margins and is also hypothesised to link boundary specification and maintenance with ligule outgrowth (Osmont *et al*., 2003, 2006). The identity of *ETA1* remains unknown. High‐resolution technologies, such as single‐cell or spatial RNA‐seq, combined with temporal analysis of mutants, will deepen our understanding of how these regulatory networks and signalling elements underlie boundary specification. Combining this knowledge with dynamic computational models will enable an understanding of how boundary specification in the leaf is established (Phase ii) and how the blade–sheath boundary is maintained (Phase iii) in a growing and deforming leaf. This dynamic information could then be combined with recent advances in synthetic biology to tailor gene expression in distinct spatiotemporal patterns (Danila *et al*., 2022), allowing precise tinkering of leaf domains across the P‐D axis, reshaping the cereal leaf.

### 3. Grass leaf growth and mechanics

Along with patterning, highly dynamic growth anisotropy occurs along the different axes to define leaf shape (Sprangers *et al*., 2020). Through differential modulation of growth patterns, diverse leaf sizes and shapes arise, for example long and narrow (Nelissen *et al*., 2012), or short and wide (Foster *et al*., 1999; Chen *et al*., 2014; Kaur *et al*., 2024), short and narrow (Durbak *et al*., 2014), or long and wide (Sun *et al*., 2017). Moreover, leaf morphology can change over development. Most, if not all, growth processes are associated with changes in hormonal balances (Nelissen *et al*., 2012; Sun *et al*., 2017; Sprangers *et al*., 2020; Robil & McSteen, 2023; Kaur *et al*., 2024), with auxin, GA, CK, and BR as main contributors. Since constitutive perturbations of hormones often result in pleiotropic effects, and multiple genes are involved in hormone biosynthesis, catabolism, perception, and responses, higher order resolution of the spatiotemporal accumulation of transcripts and metabolites is needed to formulate engineering strategies that will enable fine‐tuning. With increased sensitivity and resolution of technologies, such as spatial transcriptomics or metabolomics, or a combination thereof, coupled with the relatively large size of the cereal leaves, it is promising that hormonal distribution maps will become available in the near future (Liao *et al*., 2015; Rizza *et al*., 2017; Laureyns *et al*., 2022; Dao *et al*., 2025; Petřík *et al*., 2024; Wu *et al*., 2025).

Growth also causes internal mechanical stress signals that feedback on cell expansion, and thus affect leaf size, shape, and structure. These mechanical cues play a role during different processes of leaf growth and morphogenesis, such as leaf initiation, postinitiation organ growth and patterning, and are often localised at boundary domains and transition zones (Echevin *et al*., 2019; Hamant & Saunders, 2020; Neher *et al*., 2023). Boundary regions and transition zones are typically associated with spatiotemporal dynamics of gene expression (Maugarny‐Calès *et al*., 2019; Satterlee *et al*., 2023), hydraulic patterns (Alonso‐Serra *et al*., 2024), turgor pressure (Coussement *et al*., 2021), hormone accumulation and signalling (Nelissen *et al*., 2012; Sprangers *et al*., 2020; Han *et al*., 2022), cell wall properties (Jonsson *et al*., 2022), division orientation (Neher *et al*., 2023), and the interplay between these processes (Echevin *et al*., 2019; Neher *et al*., 2023). Knowledge of each of these mechanisms is constantly increasing due to technological improvements, such as atomic force microscopy and spatial‐omics (Richardson *et al*., 2021; Laureyns *et al*., 2022; Neher *et al*., 2023; Petřík *et al*., 2024). Multidisciplinary efforts that merge these state‐of‐the‐art technologies will highlight the inherent interplay between physics, genetics, and metabolism, building a predictable model of grass leaf development to guide precision engineering approaches.

## III. Formation of specialised structures and cell types

Grass leaves have distinctive morphological features associated with the underlying axial patterns that significantly impact productivity and yield. For example, ligule and auricles, parallel venation, Kranz anatomy, epidermal patterning, and cuticle formation collectively have unique features in the grasses and influence how the plant interacts with its environment.

### 1. Ligule and auricle formation

The ligule and auricle region displays remarkable morphological diversity in the grasses (Philipson, 1935; Kellogg, 2015a; Edson‐Chaves *et al*., 2022). Together, they serve a critical function to angle the blade away from the stem, a key agronomic trait (Tian *et al*., 2011, 2019, 2024; Kong *et al*., 2017; Wang *et al*., 2024). Work investigating genetic networks that control ligule formation has exploded alongside technological advances and genome editing (Impens *et al*., 2023; Ahmar *et al*., 2024; Lorenzo *et al*., 2024).

The SQUAMOSA BINDING PROTEIN transcription factor LG1 is a key driver of ligule and auricle formation and outgrowth. Maize, rice, wheat, sorghum, and barley *lg1* mutants having a clear blade–sheath boundary but, importantly, lack ligule and auricles, resulting in conspicuously upright leaf blades (Fig. 5a) (Moreno *et al*., 1997; Lee *et al*., 2007; Richardson & Hake, 2018; Yu, 2019; Brant *et al*., 2021; Yang *et al*., 2022). Conversely, changes in the expression of *LG1* result in abnormal leaf angles, as observed in maize and rice *increased leaf inclination1* (*ili1*) (Ren *et al*., 2020), and maize *indeterminate domain14* (*idd14*) and *idd15* mutants (Liu *et al*., 2024). ILI1 physically interacts with IDD14/15 in a protein complex that binds to the *LG1* promoter to regulate its expression (Liu *et al*., 2024). Unsurprisingly, polymorphisms that associate with *LG1* for natural variation in leaf angle have been detected in maize genome‐wide association studies (GWAS) and quantitative trait loci (QTL) mapping (Tian *et al*., 2011), highlighting the potential for modulating *LG1* expression or function in cereals to optimise leaf angle and canopy architecture. The utility of *lg1* mutant alleles in commercial breeding programmes, however, is limited (Lambert & Johnson, 1978), likely because of the severe upright blades at all nodes (i.e. canopy‐wide). By contrast, an ideal cereal canopy would be composed of plants with upright upper leaves, slightly more lax middle leaves, and more sprawling lower leaves (Tian *et al*., 2011, 2019, 2024; Kong *et al*., 2017; Wang *et al*., 2024). Uncovering weak alleles and/or networks and modules in which LG1 functions is central to identifying pathways to precisely tailor blade angle throughout plant development. Studies that leverage technological advances and germplasm resources, built on decades of careful developmental analyses, hold great promise for revealing aspects of the *LG1* network to engineer cereal architecture.

**Fig. 6:**
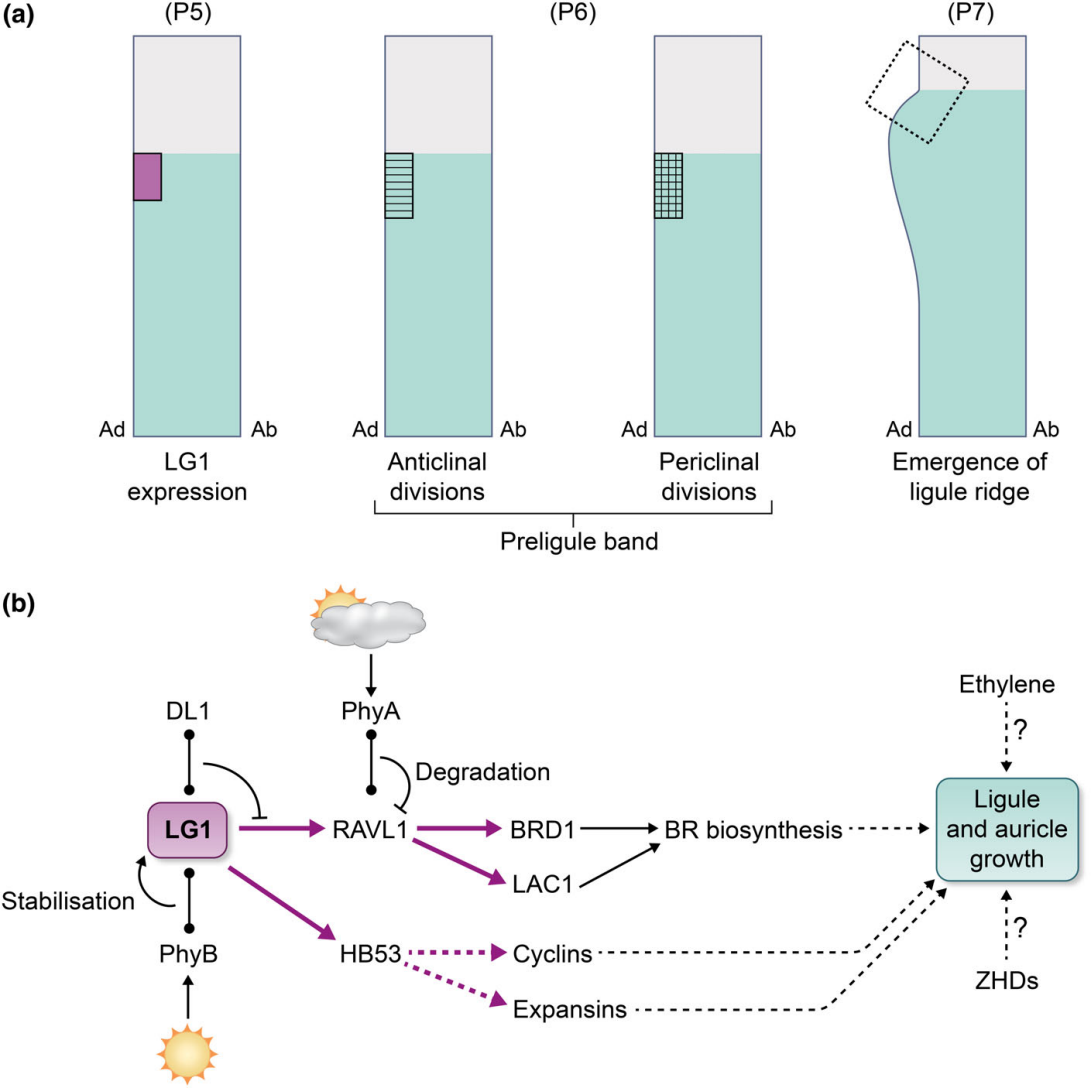
Ligule outgrowth and development. (a) Stylised cartoons of leaf longitudinal sections through the developing ligule region. Ligule outgrowth follows a series of distinct gene expression and morphological changes across plastochrons (P#). Before any morphological changes are observed, *LIGULELESS1* (LG1, magenta) is expressed in a subset of adaxial (Ad) epidermal cells at the distal end of the sheath (blue). Next, the preligule band is formed through anticlinal and periclinal divisions in the region marked by LG1 expression in the adaxial (Ad), but not the abaxial (Ab) side of the leaf. Differential growth then leads to outgrowth of the ligule ridge, with small cells in the node of the ligule (boxed region). (b) Current understanding of the potential LG1 network controlling ligule and auricle growth. If these interactions occur in the same tissues and at the same time is not currently known. Dotted ends: physical interactions between proteins. Arrows: positive regulatory interaction. Magenta arrows: positive transcriptional regulation. Blunt ends: inhibitory regulatory interaction. Solid lines: direct regulatory relationship. Dotted: indirect/ unknown regulatory relationship. Sun: high light conditions. Cloud: shade conditions. Phy: Phytochrome; DL1: DROOPING LEAVES1; RAVL1: RELATED TO ABSCISIC ACID‐INSENSITIVE3/VIVIPAROUS1‐LIKE 1; HB53: HOMEBOX PROTEIN 53; BRD1: BRASSINOSTEROID DEFICIENT1; LAC1: LEAF ANGLE ARCHITECTURE OF SMART CANOPY1; BR: BRASSINOSTEROID; ZHD: ZINC FINGER‐HOMEODOMAIN.

Consistent with its key role in driving ligule formation, *LG1* expression precedes any morphological changes at the blade–sheath boundary. In maize, *LG1* transcripts begin to accumulate in a small patch of adaxial epidermal cells in P5‐stage primordia (Johnston *et al*., 2014; Lewis *et al*., 2014). By P6, a distinct ridge of sheath epidermally derived cells, called the preligular band, which accumulates LG1 as well as PIN1a, delineates the sheath and blade molecularly and morphologically (Sharman, 1942; Becraft *et al*., 1990; Sylvester *et al*., 1990; Moon *et al*., 2013; Johnston *et al*., 2014; Neher *et al*., 2023; Satterlee *et al*., 2023). Cells within the preligular band have increased and localised anticlinal (i.e. within the plane) cell divisions, with subsequent periclinal (i.e. parallel to the plane) cell divisions marking ligule initiation and outgrowth (Sylvester *et al*., 1990; Neher *et al*., 2023; Fig. 6a). Concomitant with this process is the recapitulation of the gene expression patterns observed during organ initiation (Johnston *et al*., 2014; Satterlee *et al*., 2023). Alongside ligule outgrowth, auricles are specified from adaxial epidermal cells that sit at the boundary between the preligular band and preblade (Sylvester *et al*., 1990; Neher *et al*., 2023). Ultimately, lignified hypodermal sclerenchyma cells are formed in parallel arrays, and it is these cells that structurally support the blade (Makarevitch *et al*., 2012; Strable *et al*., 2017; Tian *et al*., 2019, 2024; Wang *et al*., 2024). How LG1 triggers this organogenic process and integrates with hormone pathways to influence blade angle remains an active area of research.

Combined data from laser‐capture RNA‐seq, QTL mapping, and CRISPR‐Cas9 editing is beginning to elucidate how LG1 controls ligule formation (Fig. 6b). Dissecting QTLs in recombinant inbred lines between maize and its wild progenitor teosinte has identified multiple downstream components, including *Upright Plant Architecture1* (*UPA1*) and *UPA2* QTL (Fu *et al*., 2019; Tian *et al*., 2019). *UPA2* is associated with a nucleotide polymorphism in a *cis*‐regulatory element (CRE) upstream of the *RAVL1* gene encoding a B3‐domain transcription factor (Tian *et al*., 2019). This CRE is bound and activated by LG1. The YABBY transcription factor DL1 can also physically interact with LG1, dampening its binding affinity to *UPA2*. RAVL1 directly targets *UPA1/BRD1*, which encodes an enzyme required for BR biosynthesis, a key hormone regulator of ligule/auricle development (Conklin *et al*., 2019; Satterlee & Scanlon, 2019; Xu *et al*., 2021; Cao *et al*., 2022; Perico *et al*., 2022; Gong *et al*., 2024; Zhang *et al*., 2024). In teosinte, DL1‐LG1 associates more strongly at *UPA2* than in maize, reducing *RAVL1* expression, and thus, lowering *BRD1* and BR levels that underpin the smaller leaf angle (Tian *et al*., 2019). Further linking LG1 with BR, *LEAF ANGLE ARCHITECTURE OF SMART CANOPY1* (*LAC1*) is a direct target of RAVL1 and is involved in BR biosynthesis (Tian *et al*., 2024). *lac1* maize plants have upright upper leaves, slightly more lax middle leaves and more sprawling lower leaves, and display improved light penetration and enhanced photosynthesis rates at higher planting densities (Tian *et al*., 2024), thus suggesting that modulation of the *LG1* network can be differentially harnessed to regulate whole‐canopy architecture across development.

Further probing the LG1‐RAVL1 network has also highlighted a link with environmental signalling. RAVL1 physically interacts with Phytochrome A (phyA) photoreceptors that accumulate in shade conditions associated with high planting density. PhyA association with RAVL1 targets it for degradation via the 26S proteasome and consequently results in lower *LAC1* expression and reduced BR levels in developing auricle cells (Tian *et al*., 2024), which potentially explains the stage‐specific phenotype of the *lac1* mutants. Conversely, at lower plant densities, PhyB photoreceptors accumulate and physically interact with LG1 to stabilise it (Shi *et al*., 2024). This allows LG1 to associate directly with the promoter of the *HOMEOBOX53* (*HB53*) gene, which encodes a transcription factor that regulates the expression of *CYCLIN* (*CYC*) and *EXPANSIN* (*EXP*) genes. This module for driving cell division and cell elongation, respectively, in the developing ligular region widens leaf angles (Shi *et al*., 2024). To further link *LG1* with shade avoidance, *LG1* and *phyC* genes are key candidates identified in a maize GWAS for the reorientation of upper canopy leaves parallel to leaves of adjacent plants (Zhou *et al*., 2024), thus highlighting a need to not only understand hormones but also the environment over developmental time.

Ectopic expression of *LG1* results in auricle formation in the maize leaf blade (Hay & Hake, 2004; Lewis *et al*., 2014), indicating that auricle formation is likely downstream of boundary specification and ligule outgrowth. This is consistent with the timing of auricle differentiation, which is relatively late in leaf development (Neher *et al*., 2023). Work in rice that tracked epidermal cell dynamics in developing auricles uncovered differences in cell number between auricle margins and medial planes (Xu *et al*., 2025), suggesting that auricle formation requires coordination between tissue layers and across the patterned axes that were established earlier in leaf development. Genes specific for auricle development have not been well characterised, although recent single nuclei transcriptomes from cells in the maize ligular region identified *EXP* genes and genes encoding bHLH30 and bHLH155 transcription factors that are upstream transcriptional activators of auricle cell differentiation (Wang *et al*., 2024).

Clear roles for hormone pathways have been demonstrated for auricle development. BR signalling, in particular, is a well‐characterised regulator involved in auricle formation, with diverse BR signalling mutants having altered auricle cell sizes (Makarevitch *et al*., 2012; Kir *et al*., 2015; Tian *et al*., 2019, 2024). Auxin has also been linked to auricle formation through analysis of rice *zhd1;zhd2* double mutants that have upright blades due to reduced cell area and cell number in lateral edges of mutant auricles (Xu *et al*., 2025). Maize *zhd1;zhd21* double mutants similarly decrease blade angle (Bertolini *et al*., 2024); however, the underlying mechanisms for this have yet to be reported. The maize ethylene biosynthesis mutant *Semidwarf3* (*Sdw3*) also promotes elongation of auricle cells that results in a wider blade angle, in contrast to reduced cell elongation in internodes (H. Li *et al*., 2020). Pleiotropic phenotypes that arise from misregulation of hormone pathways highlight the need to fine‐tune hormone circuits to regulate auricle size and subsequent blade angle, as well as the need to dissect pleiotropic regulatory circuits to enable more predictable engineering of leaf traits.

### 2. Vascular structures

The precise parallel pattern of vein formation characteristic of grasses is laid down in the early developing leaf primordium and regulated spatiotemporally. Vein development is best characterised in the C_4_ species maize, where four ranks of veins form longitudinally at specific times in leaf development. This starts with the midvein, arising from internalisation of auxin maxima in the initiating primordium (i.e. P1). Afterwards, lateral veins arise *de novo* from procambial initials in inner layers of P2/P3‐staged leaf primordia (Langdale *et al*., 1989; Carraro *et al*., 2006; O'Connor *et al*., 2014; Johnston *et al*., 2015; Robil & McSteen, 2023). Rank 1 and 2 intermediate veins are then established between laterals during P3 (Perico *et al*., 2024). Once parallel venation is set longitudinally, transverse veins form horizontally across the leaf during leaf elongation. In addition to the M‐L patterning that defines distribution and density of longitudinal veins, P‐D patterning results in only rank 1 intermediate veins extending into the sheath. Vein density, for example, is a key target for engineering C_4_ photosynthesis into C_3_ cereals (Vlad *et al*., 2024), and yet information on the precise spatiotemporal regulation of vein patterning is scant. A proposed prepattern underlies vein initiation and subsequent spacing in early‐stage primordia (Perico *et al*., 2022, 2024). In support of this hypothesis, molecular cartography (i.e. targeted multiplex *in situ* hybridisation) in maize indicates procambial initials are already arranged properly upon their accumulation of PIN1a; however, how such regular spacing in the M‐L axis during the earliest stages of leaf development manifests remains to be fully elucidated (Perico *et al*., 2024).

Veins in grass leaves are surrounded by one ring or multiple rings of mestome and/or bundle sheath cells (Sedelnikova *et al*., 2018). In rice, barley, and other C_3_ grasses, a procambial‐derived inner mestome layer encircles veins, which itself is surrounded by bundle sheath cells initiated from ground meristem (Williams *et al*., 1989; Sakaguchi & Fukuda, 2008; Kellogg, 2015b). Within these structures, additional differentiation across the Ad‐Ab axis also occurs. Bundle sheaths in barley are hypothesised to be functionally differentiated; abaxial large (L‐type) cells and plasmodesmata‐dense small (S‐type) cells encompass mestome and phloem, which are thought to aid in the movement of photoassimilates (Williams *et al*., 1989). In maize, procambial initials give rise to veins and bundle sheath cells that encircle them (Langdale *et al*., 1989). Single‐cell RNA sequencing of mature leaf blades identified two distinct clusters between the transcriptomes of smaller abaxial and larger medial bundle sheath cells (Bosabalidis *et al*., 1994; Bezrutczyk *et al*., 2021). These Ad‐Ab differences were confirmed via transgenic lines expressing translational fusions of candidate SWEET sucrose uniporters (Bezrutczyk *et al*., 2021). It is therefore important to consider key genetic network differences when engineering vein density and structure that could be impacted by attempts to modulate Ad‐Ab and P‐D leaf patterning networks.

### 3. Epidermal patterning and cuticle formation

The epidermis contains highly specialised cell types, the composition of which varies between species and spatially along leaves, impacting environmental resilience and responses. Stomatal guard cells (GCs), for example, are required for efficient gas exchange and transpiration in land plants, and the grasses have evolved companion subsidiary cells (SC) which improve gas exchange efficiency by facilitating rapid changes in stomatal aperture (Franks & Farquhar, 2006; Raissig *et al*., 2017). In addition, monocot‐specific cork and silica cell pairs are believed to improve structural support, function as mineral reservoirs (Kaufman *et al*., 1985) and may play a role in herbivore deterrence (McNaughton & Tarrants, 1983). Despite the importance of these surface features, the coordinated temporal and spatial mechanisms required to generate this fully patterned and functional epidermis with parallel columns of cell pairs, for example, silica‐cork and stomata‐SC, separated by pavement cells (Kaufman *et al*., 1985; Franks & Farquhar, 2006), are unknown.

Epidermal differentiation occurs over a developmental gradient from the base to the tip of the leaf, with asymmetric divisions along the P‐D axis separating each specialised cell with a single ‘default’ pavement cell during leaf elongation. This is coordinated with the underlying parallel venation pattern established earlier. Development of live imaging techniques and reporter lines in diverse grass species has enabled tracking of these cell lineages over time (e.g. Ashraf *et al*., 2023; Spiegelhalder *et al*., 2024) and comparisons with the eudicot model Arabidopsis in which the one‐cell spacing mechanisms are best understood (reviewed by Lee & Bergmann, 2019) have helped build a picture of the dynamic nature of epidermal patterning in grasses. While many of the core factors required for stomatal patterning, such as SPEECHLESS (SPCH), MUTE, and FAMA, are retained in the grasses (Raissig *et al*., 2017; Wang *et al*., 2019; Wu *et al*., 2019), several of the fundamental mechanisms have undergone functional divergence, potentially underpinning the distinct morphological features of the grasses. For instance, stomatal cell fate is established in a subset of protodermal cells at the proximal end of the grass leaf, but these cells do not carry out self‐renewing divisions. An asymmetric division, controlled by SPCH, directly produces one pavement cell and one guard mother cell that will go on to produce the GCs (Raissig *et al*., 2016). MUTE also has a co‐opted role in controlling SC recruitment from flanking cell files in brachypodium and maize (Raissig *et al*., 2017; Wang *et al*., 2019), and FAMA can compensate for MUTE's role in driving GC fate in brachypodium (McKown *et al*., 2023). The SCREAM orthologues have also functionally diverged in brachypodium, with BdICE1 required for stomatal initiation and BdSCRM2 implicated in GC and SC differentiation (Raissig *et al*., 2016). How these regulators relate to the underlying leaf axial patterning is still poorly understood. The ability to fine‐tune stomatal density and enhance water use efficiency is an attractive avenue for boosting crop productivity, and with improved understanding of the unique features of grass stomatal regulation, this is becoming increasingly possible. Recent studies have modulated stomatal density in rice using gene editing (Karavolias *et al*., 2023; Rathnasamy *et al*., 2023), but another intriguing approach is the application of bioactive peptides to modulate stomatal density. In brachypodium, application of EPIDERMAL PATTERNING FACTOR 2 (EPF2) peptides inhibits entry into the stomatal lineage, while co‐application of STOMAGEN peptides mitigates this effect (Jangra *et al*., 2021).

Far less is known about the regulation of other specialised epidermal cells. Plasticity in cell fate determination appears to be important to consider if we are to engineer epidermal cell fates – SQUAMOSA promoter binding protein like (SPL)10, SPL14, and SPL26 control trichome fate in maize leaves, and intriguingly, loss of these factors promotes the formation of ectopic stomatal complexes (Kong *et al*., 2021) for example. Similarly, a shared role in the patterning of stomata, prickle cells, and silica‐cork cell pairs has been revealed for the YODA‐driven Mitogen‐Activated Protein Kinase (MAPK) cascade in brachypodium and barley by regulating cell fate reinforcement after asymmetric division (Abrash *et al*., 2018; L. Liu *et al*., 2022). While our understanding of epidermal patterning in the grasses is limited, the advent of single‐cell and spatial technologies will be invaluable for unravelling the regulation and function of specific cell types. Recent single nuclei profiling in maize has already provided novel insights into the transcriptomes of GCs and SCs in developing leaves, revealing cell‐specific transcripts, which may coordinate degradation and influx of abscisic acid in SCs and GCs, respectively (Sun *et al*., 2022). Combining this information with spatial and temporal information will enable the connection between leaf patterning and growth and epidermal cell specialisation to be made.

#### Cuticle

The cuticle forms at the interface between the plant and its environment, and the primary function is to minimise water loss and protect against biotic and abiotic stresses, but it also plays a crucial role in defining organ boundaries (Ingram & Nawrath, 2017). Most of the components of the lipid‐rich cuticle are common across land plants (Kong *et al*., 2020), but there is incredible diversity in the exact composition and structure across and within species, depending on specific environmental and developmental contexts. For example, many cereal cultivars deposit a thick wax bloom consisting of glaucous wax crystals on upper leaf sheaths, stems, and inflorescences once exposed to the atmosphere, and it is related to drought tolerance (Larson *et al*., 2024).

Cuticle is deposited on the leaf epidermal surface as it develops, and the composition and structure vary along the P‐D axis as the leaf emerges from the whorl and between leaves. For instance, in maize leaf blades, smaller chain fatty acids are one of the first components to be deposited on the epidermis, followed by a rapid increase in wax esters and a gradual increase in cutin and longer chain fatty acids towards the distal tip (Qiao *et al*., 2020). The elaboration of this complex layer coincides with cuticle thickening and a decrease in cuticular permeability along the P‐D axis as the leaf matures (Bourgault *et al*., 2020). Although key enzymes in fatty acid synthesis and transport have been identified in grasses (e.g. Müller *et al*., 2023; Campoli *et al*., 2024; Zhao *et al*., 2024), how these are developmentally regulated both spatially and temporally is less clear.

Most known regulators of cuticle formation belong to either the AP2, HD‐ZIP, or MYB superfamilies, and only a handful are characterised in grasses. In the blade, the AP2 transcription factors WAX SYNTHESIS REGULATORY GENE 1 (WR1) and WR2 in rice, and WAX INDUCER 1 (WIN1) in wheat regulate wax biosynthesis (Wang *et al*., 2012; Zhou *et al*., 2014; Kong & Chang, 2018). By contrast, in barley, HvWIN1 regulates cuticular biosynthetic pathways in leaf sheaths but not blades and is essential for wax bloom formation (McAllister *et al*., 2022). The HD‐ZIP IV transcription factor RICE OUTERMOST CELL SPECIFIC GENE 4 (ROC4), negatively regulated by the activity of the E3 ubiquitin ligase DROUGHT HYPERSENSITIVE (DHS), is also required for wax synthesis in the blade and is linked to drought tolerance (Wang *et al*., 2018). The maize MYB transcription factor FUSED LEAVES1 also controls lipid accumulation in the blade by regulating genes encoding cuticular enzymes and ABCG transporters (Castorina *et al*., 2020). How these regulators interact and connect with the underlying developmental patterning is unclear. Building these networks will be crucial for understanding how the cuticle is coordinated, and therefore how best to programme ideal cuticle composition for drought resilience.

## IV. Engineering a grass leaf

Although we have a textbook view of the cereal leaf (Fig. 1c), the reality is that no single view fully captures its developmental dynamics. All leaves of one cereal plant have the same basic organisation. However, different leaves that form during the lifecycle of the plant have remarkable characteristics. In modern maize hybrids, for example, the longest leaves are typically found around the ear, whereas the uppermost leaves are more upright, shorter, and possess less sheath. This ideotype maximises incident light for photosynthesis, even at high planting density (Mantilla‐Perez & Salas Fernandez, 2017). Shifting our view of leaf development from static to dynamic is essential to build new plant varieties that are adaptable and responsive to specific environmental conditions and management practices.

Breeding efforts in recent decades have largely focused on increased planting density, which in turn increases yield per unit area (Duvick, 2005). These yield gains are associated with unintentional selection for architectural changes, including more upright upper leaves (Zhou *et al*., 2024). Intentional selection and engineering of architectural traits, guided by a deep understanding of dynamic leaf development, could therefore prove highly valuable. For example, leaf shape and width influence water use efficiency (George‐Jaeggli *et al*., 2017), and water availability varies over the plant's lifecycle. Increasing breeding programme emphasis on developing shorter stature plants, while maintaining ear height in maize, for example, will also require changes in the number, shape, and angle of leaves, depending on their position on the plant. These examples highlight the crucial relationship between fundamental understanding of plant architecture, environmental conditions, and breeding strategies to optimise plant performance.

**Fig. 7:**
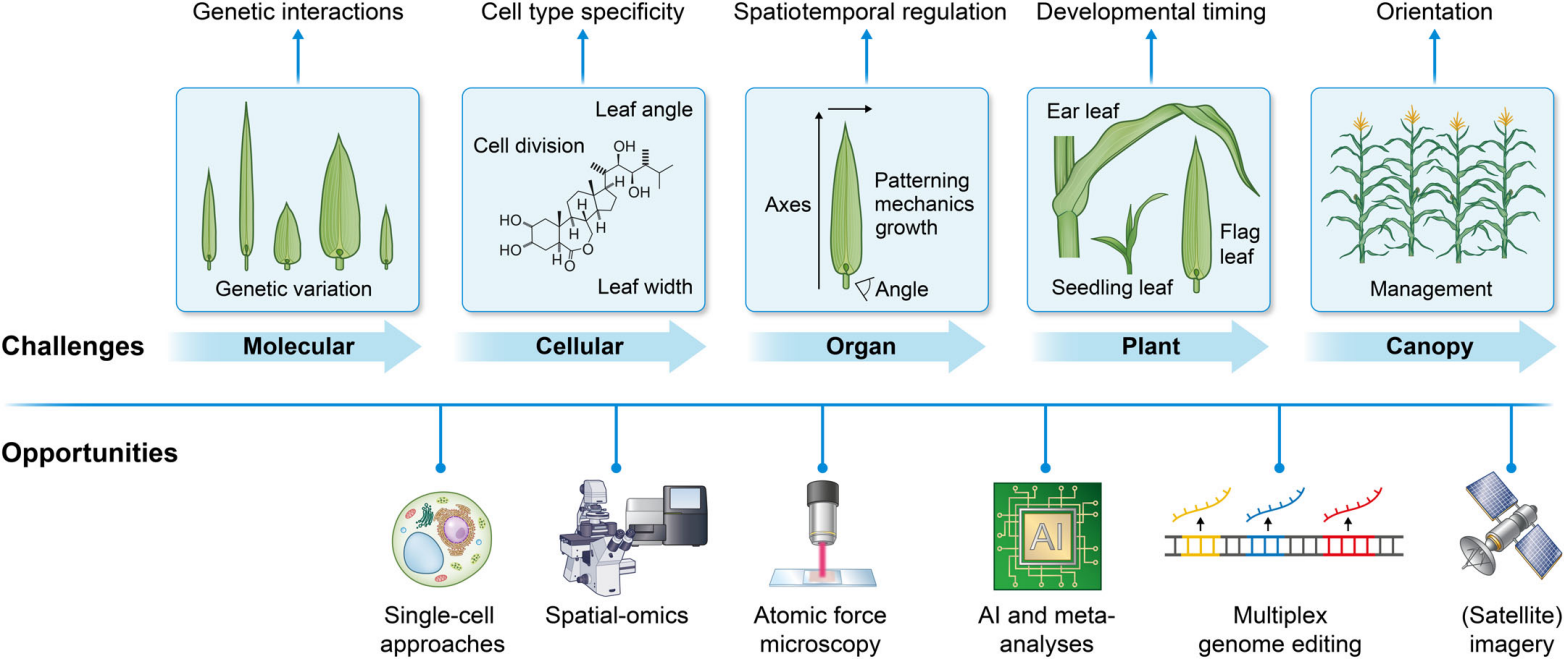
Leaf development engineering challenges and opportunities. Understanding of the dynamic mechanisms that underpin cereal leaf development, across scales (molecular: cellular: organ: plant: canopy) will be aided by recent advances in single‐cell analyses, spatial‐omics, quantitative biomechanics, artificial intelligence and meta‐analysis algorithms, multiplex genome editing and remote imaging. Figure partially created in BioRender: Hilde, Nelissen (2025) https://app.biorender.com/illustrations/67b6282fca5fffcf4c6ae549?slideId=fa7b9fd1‐8466‐45b3‐936e‐748588ab9295.

Given the indispensable nature of the cereal leaf, it is an excellent target for precision engineering. Understanding the molecular players and the developmental programmes that determine the final shape, angle, and size of leaves will open prospects for a tunable leaf where genetic variation leading to an ideotype could be introduced into elite varieties (Tian *et al*., 2019, 2024). To unlock this ability, there is an urgent need to leverage recent technological advances to develop a deeper understanding of the dynamic mechanisms underpinning leaf development and build new strategies to predictably alter leaf traits at precise times in development (Fig. 7).

There are still outstanding questions with respect to signals, molecular players, and mechanisms, emphasising that more research is needed to identify missing links, and that functional characterisation is the backdrop for understanding leaf development. In addition, the fact that the different leaves of one plant have distinct characteristics raises the question of whether a single developmental programme is executed differently at different growth stages, and if so, what signals trigger the shift in the developmental programme over time. These questions can now be addressed through leveraging the exceptional genetic resources and novel tools already demonstrated in cereals (Fig. 7). Combining these data with comparative genomics analyses between cereal species with different leaf traits – such as more or less sheathing around the stem– and evolutionary developmental approaches (e.g. Richardson *et al*., 2021; Prigge *et al*., 2025) will also elucidate potentially distinct molecular switches that control leaf shape and angle across species.

Leaf morphology is controlled by a multitude of genes, as we have illustrated in this review, and because their constitutive perturbations often result in extreme phenotypes or pleiotropic effects, there is a need to calibrate the expression of multiple genes simultaneously to achieve desired changes in leaf size and shape. To understand cell‐type specificity, relate dynamic transcriptional changes, identify novel players, and even cell types, single‐cell/nuclei analysis is revolutionising the field by providing high‐resolution insights into cellular heterogeneity and complexity. To complement high‐resolution transcriptomics with tissue context, spatial‐omics technologies (Laureyns *et al*., 2022) map how molecular mechanisms are organised and could be optimised. Rapidly emerging (multi‐)omics approaches (Marand *et al*., 2021; Baysoy *et al*., 2023; Yu *et al*., 2023; Horn & Chapman, 2024; Montes *et al*., 2024; Wu *et al*., 2024; Yin *et al*., 2024) on low‐input and *in situ* samples will drive the identification of novel molecular players and also further inform engineering approaches to design the cereal leaf. To capture the dynamic properties of such a tunable system and to predict its responses, experimentation supplemented with modelling (Richardson *et al*., 2021) and machine learning approaches (Coleman *et al*., 2024; Cui *et al*., 2024; Yang *et al*., 2024) are needed to translate the wealth of single‐cell and spatial‐omics data into testable hypotheses. Using multiplex genome‐editing approaches (Impens *et al*., 2023), combinations of genome edits can be screened for particular parameters of a leaf ideotype. Currently, all these technologies are in hand and validated in plants, often even in cereal leaves. Therefore, by coming together and combining expertise, including developmental biology, the community is in a prime position to harness such complementary disciplines to demonstrate the feasibility of achieving the ‘tunable leaf’ specific to application.

## Competing interests

None declared.

## Disclaimer

The New Phytologist Foundation remains neutral with regard to jurisdictional claims in maps and in any institutional affiliations.

## Supporting information


**Table S1** Representative list of cloned cereal mutants with defects in leaf shape, size, or arrangement.
Please note: Wiley is not responsible for the content or functionality of any Supporting Information supplied by the authors. Any queries (other than missing material) should be directed to the *New Phytologist* Central Office.
